# *Mycobacterium tuberculosis* thymidylate synthase (ThyX) is a target for plumbagin, a natural product with antimycobacterial activity

**DOI:** 10.1371/journal.pone.0228657

**Published:** 2020-02-04

**Authors:** Apurba Sarkar, Shreya Ghosh, Rahul Shaw, Madhu Manti Patra, Fatema Calcuttawala, Noyonika Mukherjee, Sujoy K. Das Gupta

**Affiliations:** Department of Microbiology, Bose Institute, Kolkata, India; Institut de Pharmacologie et de Biologie Structurale, FRANCE

## Abstract

Plumbagin derived from the plant *Plumbago indica*, known as Chitrak in India, is an example of a medicinal compound used traditionally to cure a variety of ailments. Previous reports have indicated that it can inhibit the growth of *Mycobacterium tuberculosis* (Mtb), the causative agent of the deadly disease TB. In this investigation, we provide an insight into its mode of action. We show here that a significant mycobacterial target that is inhibited by plumbagin is the enzyme ThyX, a form of thymidylate synthase, that is responsible for the synthesis of dTMP from dUMP in various bacterial pathogens, including Mtb. Using a purified preparation of the recombinant version of Mtb ThyX, we demonstrate that plumbagin, a 2,4 napthoquinone, but not lawsone, a structurally related medicinal compound, inhibits its activity *in vitro*. We also show that the intracellular [dTTP]/[dATP] ratio in *Mycobacterium smegmatis* (Msm) cells decrease upon treatment with plumbagin, and this, in turn, leads to cell death. Such a conclusion is supported by the observation that over-expression of *thyx in* the plumbagin treated Msm cells leads to the restoration of viability. The results of our investigation indicate that plumbagin kills mycobacterial cells primarily by targeting ThyX, a vital enzyme required for their survival.

## Introduction

*Mycobacterium tuberculosis* is the causative agent of the deadly disease TB, which claims nearly 2 million lives yearly worldwide [1]. Although anti-TB drugs have been in existence for many years, the disease continues to be prevalent. The primary reason behind this is the emergence of drug-resistant strains of Mtb [2]. Thus, although several drugs are available, the quest for newer and more effective ones continues.

Nucleotide metabolism plays a crucial role in the survival of any organism, mycobacteria, in particular [3]. In one of our previous studies, we have reported that ectopic expression of a phage D29 gene encoding a ribonucleotide reductase led to severe impairment of mycobacterial growth and that the adverse effect was found to be due to the induction of a phenomenon commonly known as thymine less death [4]. Mycobacteria possess genes encoding two different versions of thymidylate synthases, one of which is ThyA, and the other is ThyX [5]. ThyA is ubiquitous and found in all forms of life, whereas ThyX is selectively present in a limited group of bacteria, many of which are pathogenic [5]. ThyX is considered as a legitimate drug target as it is not found in humans [6]. The fundamental difference between the two thymidylate synthases is that whereas the former functions in a dihydrofolate reductase (DHFR) dependent manner, the latter does not. ThyX is a flavoenzyme that uses the reduced form of FAD to deliver reducing equivalents to dUMP, whereas, at the same time, the enzyme transfers a methylene group from methylenetetrahydrofolate to dUMP resulting in the formation of dTMP [7]. NADPH acts as the donor of reducing equivalents to the enzyme-bound FAD, and thus, the activity of ThyX can be measured by monitoring the oxidation of NADPH spectrophotometrically. In our laboratory, we have worked with the mycobacteriophage D29 derived ThyX (Gp48) and characterized its properties [8]. In another laboratory, the Mtb version of ThyX has been studied in details [9]. In Mtb, ThyX functions as an essential enzyme. The deletion of the ThyX gene results in lethality, and therefore this enzyme is undoubtedly an excellent candidate for being developed into a drug target [10,11].

The merits of using traditional medicine to combat TB have been documented in the literature in the past [12], [13]. Although many compounds isolated from medicinal plants have been reported to have anti-tubercular activity, their precise mode of action is not known. Previously, an attempt was made to find naturally occurring compounds that block the activity of *Paramecium bursaria* Chlorella virus-1 PBCV-1 ThyX [10]. By analyzing the extracts of the medicinal plant *Diospyros maritima*, a compound, 2 -bromo, 5-hydroxy-1,4-naphthoquinone was isolated, which could inhibit the activity of *Helicobacter pylori* (Hpy) ThyX[14]. The information thus obtained was used to perform predictive modeling to identify novel [13] compounds related to 1–4 naphthoquinones that could modestly inhibit the growth of Mtb apparently by blocking the activity of not just ThyX but DNA gyrase as well [13]. In another investigation, fluorinated pyrimidine analogs were identified that could inhibit both ThyA as well as ThyX [9].

Due to the apparent failure of modern-day drugs to combat TB, efforts are on to have a relook into traditional medicine, to explore the possibility of discovering more effective anti-TB compounds [12],[15]. In traditional medicine, we find references to several plant-derived products that have significant medicinal value. One such product is plumbagin (5-hydroxy-2-methyl-1,4- naphthoquinone). The most important sources of plumbagin are plants belonging to the Plumbaginaceae family, one of which is *Plumbago indica*, locally known as Chitrak. There are many reports which attest to its ability to act as a therapeutic agent against a host of diseases, which includes cancer [16], [17]. Plumbagin also can inhibit Mtb growth [18] [19] *in vitro*, and therefore, it has the potential to be an anti TB drug. In this study, we have chosen to investigate the mechanism by which plumbagin inhibits mycobacterial growth. Besides, we have incorporated in our study another naphthoquinone known as lawsone (2-hydroxy 1, 4- naphthoquinone). Lawsone is found in a medicinal plant colloquially known as Henna in this part of the world. In particular, we addressed the issue of whether these compounds can act as anti-mycobacterials by inhibiting ThyX. The results of our investigations indicate that plumbagin but not lawsone inhibits mycobacterial growth and that this effect is primarily due to its ability to inhibit ThyX.

## Materials and methods

### Bacterial strains and plasmids

*E*.*coli* strains XL1-Blue and BL21 (DE3) were used for gene cloning and expression purposes. Overexpression in *E*.*coli* BL-21(DE3) cells was done using the pET28a (Novagen) vector system. For expression in mycobacteria, the vector pLAM12 [20] was used. The gene to be expressed was PCR amplified and cloned within the Nde1 and Nhe1 sites of the vector. Induction of gene expression from mycobacterial vectors was done using the reagent acetamide. For mycobacterial experiments, the fast-growing saprophyte *Mycobacterium smegmatis* (Msm) was used. However, in some cases, Mtb (Ra) was used, which is an avirulent version of the pathogenic Rv strain.

### Chemicals and reagents

Restriction endonucleases/DNA-modifying enzymes were obtained from Thermo Scientific or New England Biolabs. Luria-Bertani broth and Middlebrook 7H9 (MB7H9) broth were obtained from Himedia and Difco (BD), respectively. Plumbagin, (5-hydroxy-2-methyl-1,4-naphthoquinone), and lawsone (2-hydroxy-1,4-naphthoquinone) were purchased from Sigma-Aldrich chemicals. Other chemicals required for routine molecular biology work and biochemical assays were purchased from Himedia Ltd. All other chemicals/reagents for protein purification and analysis were of the highest purity and obtained from Sigma, SRL, or Merck (India). [5-^3^H] dUMP (Sp. Activity 22 Ci/mmol) was purchased from Moravek Inc.

### Growth inhibition assay

In most of the experiments, growth inhibition assays were done by first growing mycobacterial cells in MB7H9 medium to early log phase and then adding the inhibitor at the desired concentration. The cultures were then incubated further for either 24 hrs, or as mentioned, followed by the determination of optical densities at 600 nm (OD_600_). For examining cell viability, aliquotes were removed from the culture tubes and plated on MB7H9 agar. Colonies that appeared subsequently were counted. Viable counts were expressed in the units of colony-forming units per ml (CFU/ml). Determination of the Minimum Inhibitory Concentration (MIC) and Minimum Bactericidal Concentration (MBC) was done by modifying the above protocol as follows. Culture tubes containing MB7H9 growth medium plus graded doses of the test compound were inoculated with either Msm or Mtb (Ra), at a density of ~ 10^5^ CFU/ml. The cells were grown for 2–3 days in the case of Msm and 7–9 for Mtb. Growth monitoring was done by either determining the OD_600_ (visible growth) or CFU/ml. The minimum concentration of the test compound at which no visible growth was detected was considered as MIC. Similarly, the minimum concentration at which the depletion of CFU/ml occurred by over 99% was regarded as the MBC.

In the case of Mtb, resazurin microtiter assay (REMA) was also performed for MIC determination [21] [22]. For this assay, the redox dye resazurin was added to the cultures at the end of the incubation period. In REMA, cell viability is indicated by the development of a pink color due to the reduction of resazurin. The minimum concentration of the inhibitor that prevents the appearance of pink color is considered as the MIC.

For comparing the inhibitory effect of plumbagin with that of the well-known anti TB drug rifampicin, the agar cup assay was performed. MB7H9 nutrient agar plates were prepared aseptically by seeding 100 μl of Msm inoculum of O.D_600_ ~1.5 to the 15 ml of nutrient agar media. The plates were allowed to solidify. With the help of a cork borer, wells were drilled into the agar plates. 50μl of each inhibitor was dissolved and diluted with DMSO and added into the wells, with increasing concentration (5–25 μg/ml), except for one well, which contained the solvent without any drug. The plates were prepared in triplicate and incubated at 37± 0.5°C for 24 hrs. The diameter of the zone of inhibition was measured by taking the clear zones formed without the bacterial lawn. The assay was analyzed using linear fitting of the squared diameter of the inhibition zones to the natural logarithm of inhibitor concentration [23]. The MIC was determined as the anti-log value of X- intercept of a linear regression of the squared size of the inhibition zones plotted against the natural logarithm of the antibiotic concentration [24].

### Expression in *E*.*coli* and protein purification

For the purification of the MtbThyX, the gene encoding it (Rv2754c) was cloned into the six His-tagged plasmid pET28a and expressed in *E*.*coli* BL-21(DE3) strain. Over-expression of the recombinant 6X His tagged Mtb ThyX that is produced from the recombinant plasmid and its purification were performed substantially in the same way as was earlier done for the mycobacteriophage D29 ThyX [8].

### Enzyme inhibition assay

The activity of ThyX was determined by assaying for its ability to oxidize the reductant, NADPH. NADPH oxidase assays were performed as reported earlier [8]. To accomplish these assays, 40μM dUMP, and purified recombinant ThyX (0.24 μM) were added to the assay buffer comprising 50mM Tris-HCl pH 7.4, 1mM MgCl_2_, 10% glycerol, 10μM FAD, and mixed in a cuvette. Reactions were started by the addition of 200μM NADPH. The decrease in absorbance at 340 nm due to NADPH oxidation was monitored continuously over time. From the traces obtained, the initial velocities were determined. The values obtained were plotted against inhibitor concentration. Statistical analysis was done using GraphPad Prism version 5.01.

Thymidylate synthase assays were also performed using the tritium release method [7] [8]. The activity was measured by monitoring the amount of tritium [^3^H] transferred to water from [5-^3^H] dUMP after completion of the reaction [7]. A standard reaction mixture (100μl) contained 50mM Tris-Cl (pH 7.4), 200μM 5,10 CH_2_THF, 200μM NADPH, 60μM FAD 1mM MgCl_2_ and different concentrations (10–40 μM) of radiolabeled dUMP (Specific activity 1.7357 Ci mmol-1). The activity corresponding to an assay performed with all the components except for folate was considered as blank. The reaction, which was started with the addition of 20μg of purified proteins, was allowed to proceed for 3 mins, after which it was stopped by the addition of 300μl of 100mg/ml activated charcoal suspension containing 2% TCA, to remove the unused radiolabeled substrate. Adsorption on charcoal was done at room temperature for around 2–3 hrs. followed by centrifugation at 12000 rpm for 20 min to pellet down the charcoal. Radioactivity in the supernatant was determined by a liquid scintillation counter. Enzymatic activity of the protein was expressed as nmol min^-1^mg^-1^. The activities obtained were plotted against substrate (dUMP) concentration for the determination of K_m_ and V_max_. The data points were fitted to a Michelis-Menten type equation. For the determination of IC50, activity was determined at different concentrations of inhibitor followed by best-fit analysis. Curve fitting and data analysis were performed using Graph Pad Prism software.

### Isolation and analysis of the nucleotide pool

The growth of Mycobacterial cells and induction for gene expression were done as mentioned above. The cells were grown to an exponential phase, collected at specified time intervals, and poured into tubes containing 1M formic acid. The tubes were immediately frozen in liquid nitrogen and stored at -80°C. The frozen cell samples were thawed at 37 ± 0.5°C for 30 min and immediately placed in an ice bath with mild vortexing at intervals of ~30min. Thawed cells were centrifuged at 7000 X *g* for 10 mins. The supernatant was collected and dialyzed using a 100 Da cut off dialysis tubing (Spectrum), against 100 volumes of deionized water overnight. The dialysate was then subjected to ESI-MS spectral analysis using the XEVO G2 XS Q-TOF mass spectrometer equipped with an ESI source (Waters) operating in the negative ESI mode with a flow rate of 5μl/min.

## Results

### Plumbagin, but not lawsone inhibits ThyX activity

**Fig 1:**
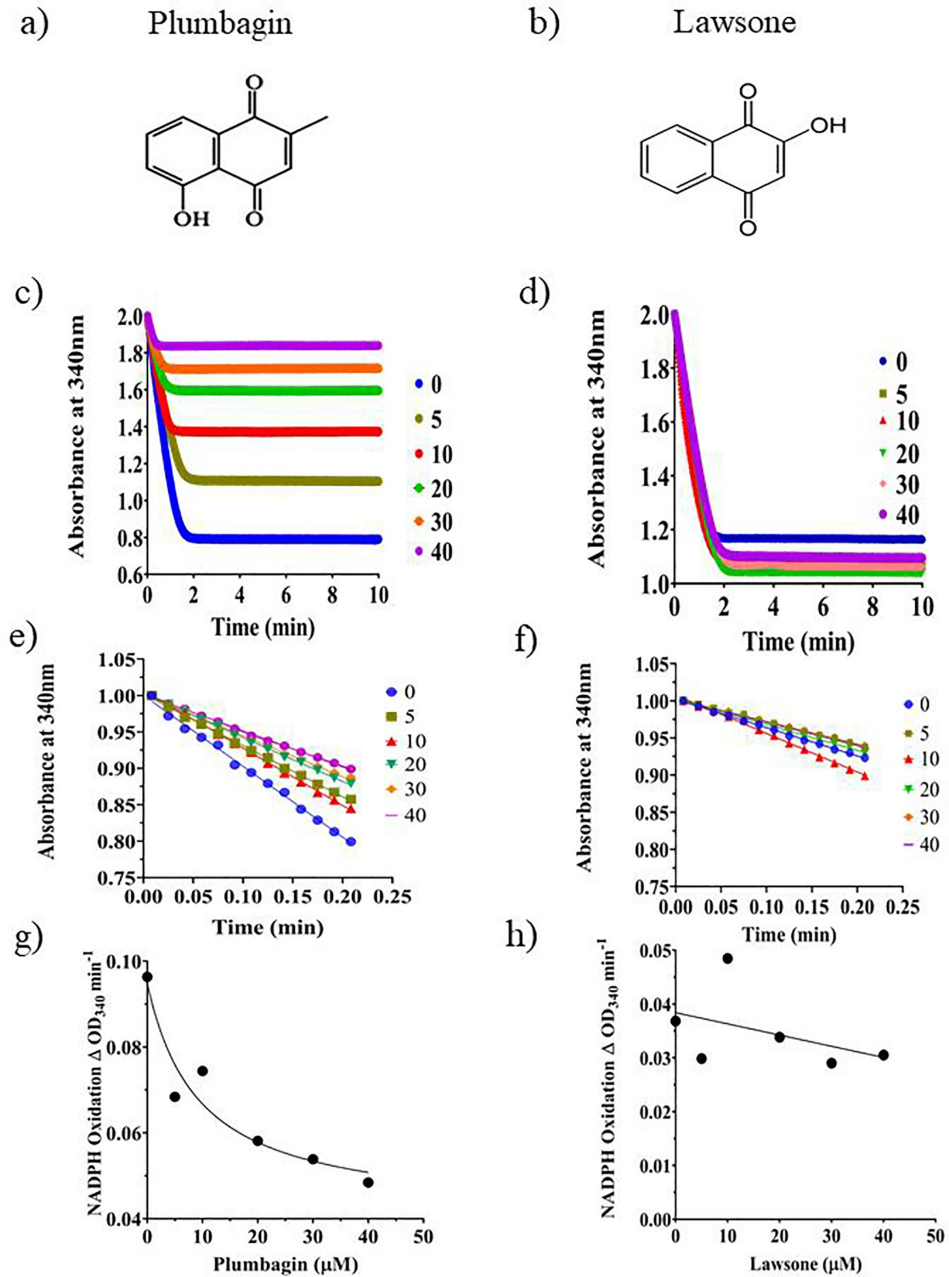
Effect of the addition of the two naphthoquinones, plumbagin and lawsone (a and b) on the dUMP dependent NADPH oxidase activity of MtbThyX (c-f) [25]. The NADPH oxidase activity was assayed by measuring the time-dependent decrease in OD_340._ The assays were carried out either in the absence or presence of different concentration (μM) of either plumbagin (c,e) or lawsone (d,f). The initial velocities derived from (e and f) obtained were plotted against inhibitor concentrations (g,h).

In a previous study, it was reported that 1, 4 naphthoquinones, particularly those with substitutions at their 5ʹ positions, can fit into the active sites of the PBCV-1 as well as HpyThyX enzymes and by doing so inhibit their activities [10], [14]. Based on this observation, we speculated that being a 1, 4 naphthoquinone, plumbagin should bind to ThyX and inhibit it. To examine the possibility that plumbagin acts as an inhibitor of Mtb ThyX, a recombinant version of the enzyme was overproduced in *E*. *coli* and purified to near-complete homogeneity (S1 Fig). The homogeneous preparation of Mtb ThyX thus obtained was then used for inhibition experiments using the NADPH oxidase assay method [10]. Increasing concentrations of plumbagin were added to a reaction mixture in which dUMP, NADPH, and ThyX were all present. The NADPH activity was then monitored spectrophotometrically by measuring the time-dependent decrease in the absorbance at 340 nm. The results of such an assay (Fig 1C) revealed that, indeed, plumbagin (Fig 1A) was capable of inhibiting the activity of the enzyme in a dose-dependent manner. The same effect, however, could not be demonstrated (Fig 1D) in case of lawsone (Fig 1B) which is also a hydroxy l, 4-naphthoquinone, but with its hydroxyl substitution located at a different position, 2 to be precise, as compared to that of plumbagin in which case it is 5. The results indicate that plumbagin but not its close relative lawsone inhibits the activity of the Mtb ThyX. The initial velocities derived from Fig (1C and 1D) were plotted against inhibitor concentration. In the case of plumbagin (Fig 1E), the velocities declined in a concentration-dependent manner (Fig 1G). Curve fitting of the values obtained to an equation of the type Y = Bottom + (Top-bottom)/(1+X/IC50) led to the determination of IC50 of about 9 μM for plumbagin. In the case of lawsone (Fig 1D, 1F and 1H), however, no inhibition was observed.

### Determination of K_i_ of plumbagin for ThyX

After obtaining an initial lead regarding the inhibitory effect of plumbagin on ThyX, we performed tritium release assays to determine the extent to which plumbagin inhibits the ability of ThyX to convert dUMP to dTMP. The amount of tritium released from [5-^3^H] dUMP in such assays is considered to be directly proportional to the amount of dTMP formed from dUMP [5].

**Fig 2:**
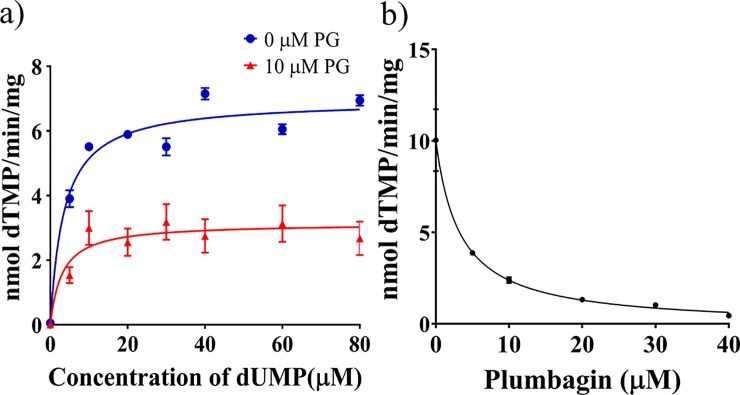
a) Determination of K_m_, V_max_, and IC_50_. Substrate (dUMP) saturation experiments, were performed, using the ^3^H release method, either in the absence of plumbagin (0 μM PG) or in its presence (10 μM PG) using Mtb ThyX. The concentrations of the co-substrates, NADPH and methylenetetrahydrofolate were 200 μM in each case. Considering the K_m_ values of ThyX (S5 Fig) for NADPH and methylene tetrahydrofolate, 35.22, and 6 μM respectively, the extent of saturation achieved was 86% for the former and 97%, latter. Curve fitting and derivation of K_m_ and V_max_ were done using the Michelis-Menten equation with the help of GraphPad Prism software. b) For the determination of IC_50_, the activity of Mtb ThyX was assayed in the presence of increasing concentrations of plumbagin (inhibitor) using a saturating concentration of dUMP (40 μM). Curve fitting was done using the formula, Y = Bottom +(Top-Bottom)/ (1+X/IC_50_), in which Y is the activity of the enzyme at any given inihibitor concentration, X. Top and bottom are plateaus in the units of the Y axis. Each data point represents the mean of three technical replicate experiments ±standard deviation. In some cases the error bars are not visible as they are too small compared to the size of the symbols.

Using this assay, we performed substrate saturation experiments to determine the K_m_ and V_max_ of ThyX for dUMP in the presence and absence of plumbagin (Fig 2A). The results indicate that the K_m_ and V_max_ for Mtb ThyX are 3.526±0.68 μM and 6.9±2 nmol/min/mg, respectively (Fig 2A, Table 1). Following the addition of plumbagin, we observed inhibition of activity. There was no significant change in K_m_, although the V_max_ decreased by about half following the addition of 10 μM plumbagin (Table 1). The results indicate that the inhibition is most likely to be of the non-competitive type for the substrate dUMP. We have estimated the IC50 value for the inhibition caused by plumbagin to be 3.315 μM (best fit in the range between 1.3 to 4.7) (Fig 2B). From the equation of non-competitive inhibition ($Vmax\;inh=Vmax/(1+\frac{I}{Ki})$, a K_i_ value of 8.21 μM was derived.

**Table 1:** Enzymological attributes of Mtb ThyX.

Substrate/inhibitor	K_m_ (μM)	V_max_ (nmol/min/mg)	IC_50_(μM)	K_i_ (μM)
dUMP	3.526±0.68	6.954±0.21	-	-
dUMP (in the presence of 10μM plumbagin)	3.137±0.2969	3.038±1.944	-	-
Methylenetetrahydrofolate	6.467	2.279	-	-
NADPH	35.22	3.231	-	-
Plumbagin (NADPH oxidase assay)			9.0	-
Plumbagin (3-H release assay)	-	-	3.315	8.21

### Plumbagin inhibits mycobacterial growth

**Fig 3:**
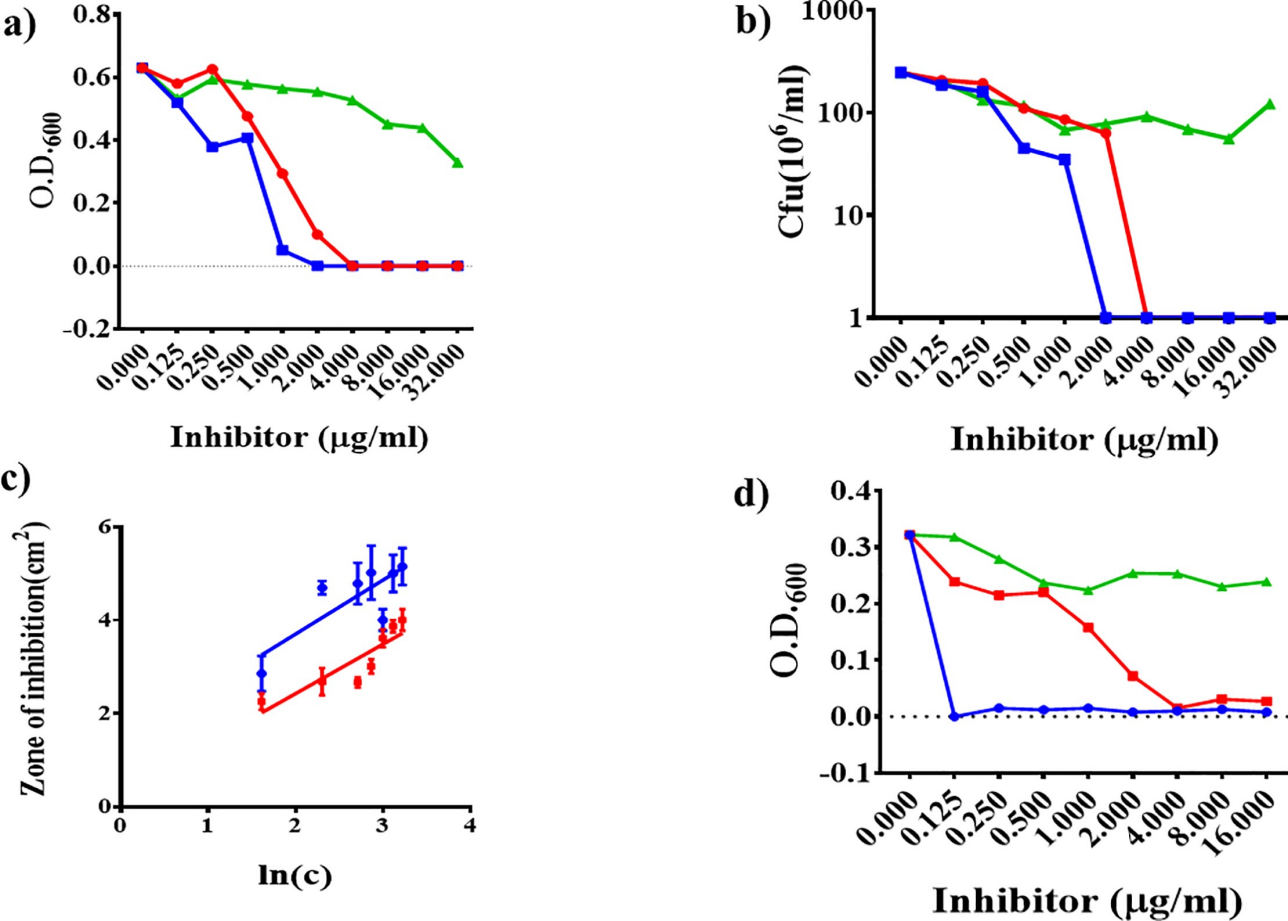
Comparison of mycobacterial growth inhibitory effects of plumbagin, lawsone, and rifampicin. Growth of mycobacteria either Msm (a and b) or Mtb (d) was monitored by measuring either visible growth (OD_600_) (a and d) or in the case of Msm (b) additionally by colony counting (CFU/ml). The MICs of plumbagin and rifampicin were also determined and compared by performing agar diffusion assay using Msm (c). Error bars in (c) represent the standard deviation from the mean of three replicate assays. The color codes are green, red, and blue for lawsone, plumbagin, and rifampicin, respectively.

The anti-mycobacterial property of plumbagin was then examined by performing growth inhibitory assays. We executed these assays by inoculating MB7H9 growth medium with Msm at a density of ~ 10^5^ CFU/ml, followed by the addition of the inhibitors, plumbagin, rifampicin, and lawsone, at the concentrations indicated (Fig 3A and 3B). Growth was monitored by determining the OD_600_ (Fig 3A) as well as viable counts (CFU/ml) (Fig 3B). The results indicate that whereas, as expected, lawsone did not affect, both rifampicin and plumbagin inhibited Msm growth in a dose-dependent manner, the MIC (and also MBC) values being 2 and 4 μg/ml, respectively. Thus we find that plumbagin is moderately (about 50%) less effective as compared to rifampicin. The inhibitory potential of plumbagin was also compared to that of rifampicin by performing agar cup assays (Fig 3C). From the slope of a best-fit line that connects the data points obtained by plotting the mean squares of inhibition diameters against the log of inhibitor concentration curves [23], it becomes apparent that plumbagin functions as an effective inhibitor of mycobacterial growth. However, consistent with what we observed in the case of the broth-based assays, compared to rifampicin, plumbagin appears to be about two-fold less effective. The estimated MIC from the agar cup assays was found to be 0.3011 and 0.7454 μg/ml for rifampicin and plumbagin, respectively.

The effect of plumbagin, lawsone, and rifampicin on the growth of Mtb (Ra) was investigated both spectrophotometrically as well as by performing REMA (Fig 3D and Table 2). As in the case of Msm, plumbagin, but not lawsone, inhibited the growth of Mtb(Ra), the MIC being the same, 4 μg/ml. Hence, the sensitivity of Mtb(Ra) towards plumbagin is the same as that of Msm. In the case of rifampicin, however, we found that the MIC for Mtb (<0.125 μg/ml) was at least 20 times less in comparison to that for Msm (1–2 μg/ml). Thus Mtb (Ra) is more sensitive to rifampicin as compared to Msm, an observation that is consistent with earlier reports [26] [27]. The results of these investigations confirm that plumbagin but not lawsone can effectively inhibit mycobacterial growth, the MIC being somewhere in between 2 and 4 μg/ml.

**Table 2:** MIC/ MBC values (μg/ml) of inhibitors reported in this study.

Inhibitor	Organism				
	Msm			Mtb (Ra)	
	MIC	MIC (Agar Cup)	MBC	MIC	MIC (REMA)
Plumbagin	4	0.74	4	4	4
Lawsone	>32	>32	>32	>16	>16
Rifampicin	2	0.3	2	<0.125	<0.125

### Nucleotide pool imbalance induced by plumbagin

**Fig 4:**
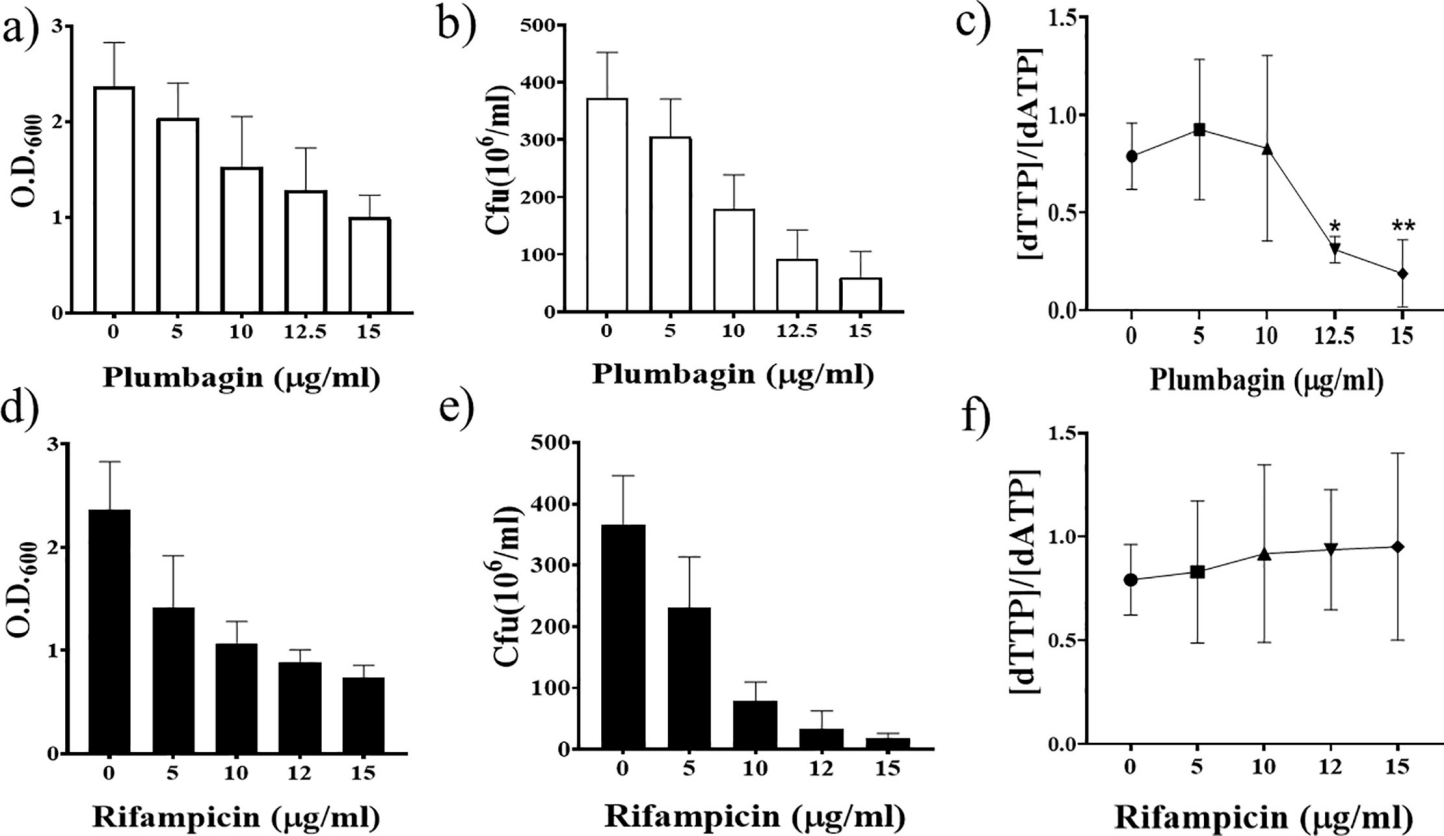
Effect of plumbagin (a-c) and rifampicin (d-f) on cell viability and [dTTP]/[dATP] ratio. Survival after plumbagin and rifampicin treatment at the doses mentioned was measured by either OD_600_ (a and d) or CFU/ml (b and e). The corresponding [dTTP]/[dATP] ratios derived by performing mass spectrometric analysis are shown in (c) and (f). The experiments were performed five times. The complete dNTP profiles from which these ratios were derived are presented in S2 Fig. The results are presented as mean ± SD (standard deviation). Significant differences in the [dTTP]/[dATP] ratios of the treated samples relative to the untreated ones are marked by asterisks, * for p = 0.0104 and ** for p = 0.0078. Significance levels (p values) were determined by performing paired t-tests using Graph Pad Prism software.

To examine whether plumbagin inhibits the activity of ThyX, and thereby induces dTTP deficiency, we first cultured Msm cells to OD_600_ of approximately 1. Then plumbagin was added at various concentrations to aliquotes of the culture in the range between 5 to 15 μg/ml and incubated at 37 ±0.5°C for 6 hrs. The levels of all the four dNTPs in the plumbagin treated as well as untreated cells were subsequently examined by extracting them from the grown cultures (Fig 4C and S2 Fig). We also determined the optical densities (OD_600_) of the cultures, as well as the number of CFUs present in them per ml after 6hrs. incubation. The results indicate that both the optical densities, as well as CFU counts, declined with increasing drug concentration (Fig 4A and 4B).

When we looked into the dNTP profiles, we observed that in the case of cells treated with relatively high doses of plumbagin (12.5 and 15 μg/ml), the level of dTTP was lower than that of dATP (S2 Fig). In contrast, in the cultures where we added less than 12.5 μg/ml plumbagin, the corresponding differences were almost non-existent. Overall the results (Fig 4C) indicate that in the case of the cells treated with lethal doses of plumbagin (≥12.5 μg/ml), the [dTTP]/[dATP] ratios were significantly less as compared to those that were untreated. A direct relationship between the decrease in optical densities and CFU counts one hand, and the lowering of the [dTTP]/[dATP] ratio on the other was thus observed. This relationship is not a non-specific one, as we did not encounter a similar decrease in the [dTTP]/[dATP] ratio when the unrelated anti-mycobacterial drug rifampicin, was used (Fig 4D–4F).

### Expression of the Mtb ThyX gene in Msm confers plumbagin resistance

The results presented above indicate that the intracellular level of dTTP was adversely affected upon plumbagin treatment due to inhibition of ThyX. If that be so, we hypothesized that the ectopic expression of a gene for ThyX should result in the reversal of the growth retarding effect of plumbagin. To test this hypothesis, we expressed the Mtb ThyX gene, in Msm from an acetamide inducible vector pLAM12 [20] and then examined whether the cells become resistant to plumbagin.

The experiment was performed by initially growing the cells to early log phase, followed by the addition of acetamide, the inducer, and incubation for another three hours. We then added plumbagin at various concentrations ranging from 5 to 25 μg/ml to the cultures followed by incubation overnight at 37 ±0.5°C. The next day we monitored the optical densities followed by plating to determine the viable counts (CFU/ml). The results obtained indicated that the optical densities of the plumbagin treated samples were less as compared to the untreated ones. Contrary to expectations, expression, or overexpression of Mtb *thyX* in the treated cells, had no significant impact on the optical densities of the cultures (S4 Fig). The lack of any observable effect was unlikely due to a defect in the expression system as we could detect the presence of abundant ThyX peptides in the extracts of these cells by performing an LC-MS/MS proteomic analysis (S3 Fig).

**Fig 5:**
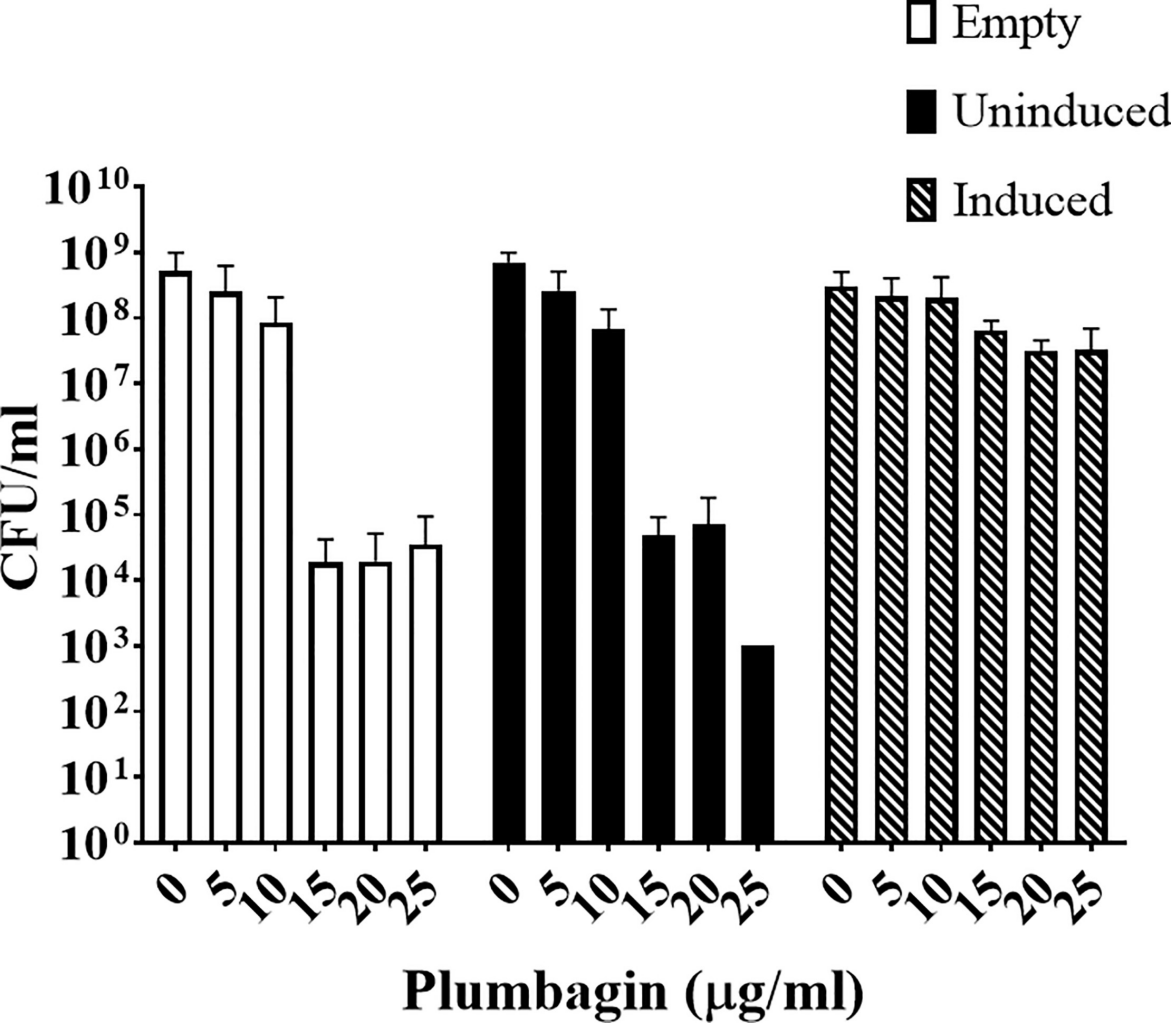
Effect of overexpression of the gene for Mtb ThyX from the inducible vector pLAM12 on the survivability of Msm cells treated with different doses of plumbagin as indicated. Survivability was measured by counting the CFUs present per ml in the cultures in which ThyX gene expression was either induced or uninduced after incubation for 24 hrs at 37 ±0.5°C. An empty vector control was also included. The actual CFU values are presented in the S1 Table.

The results obtained in the case of the CFU counts were, however, intriguing (Fig 5 and S1 Table). We found, that as expected plumbagin treatment (> 10 ug/ml) resulted in a four to five log reduction in viable counts in case of cells that were either not expressing (empty vector) or expressing the thyX gene at a basal level. But, this was not the case with cells overexpressing the gene. The considerable reduction in viability due to plumbagin treatment by over 10^4^ fold observed in the case of cells expressing *thyX* at a less than optimal level (Fig 5, empty vector and uninduced) was not evident in those that were overexpressing it (Fig 5, induced). We observed that in the case of these cells viability counts increased by at least 1000 fold relative to either the un-induced or the empty vector controls. Thus overall, it appears to us that overexpression of *thyX* resulted in a substantial protection against the bactericidal, though not the bacteriostatic, effect of plumbagin.

## Discussion

In our laboratory, we have been using mycobacteriophages as tools to discover new drugs for TB. Our investigations over the years have led us to the discovery that expression of a mycobacteriophage D29 gene, gene *50*, which codes for a class II ribonucleotide reductase in *M*. *smegmatis* results in cell death [4]. While investigating how Gp50 caused growth retardation, we found that overexpression of this gene in mycobacteria leads to the induction of thymidylate deficiency. From the observations made in that study, we hypothesized that any small molecule that brings about thymidylate deficiency should be able to function as an anti-mycobacterial agent.

Considering that there are reports of 1,4 naphthoquinones class molecules acting as ThyX inhibitors [14], we attempted to investigate whether plumbagin and lawsone, both being 1,4 naphthoquinones, do the same. We focused our attention on these compounds, as there are reasons to believe that they act as anti-TB agents. In the case of plumbagin, in particular, we found several references that testify to its ability to act as an anti-TB drug [18], [19]. In the case of lawsone, there is no direct demonstration to this effect, although there is a report which states that extracts from the Henna plant (*Lawsonia inermis*), which is the natural source of lawsone, has substantial anti-TB activity [28]. Since in an earlier investigation, it was reported that the 2 hydroxy derivatives of naphthoquinones are potent inhibitors of ThyX [13], therefore we expected that lawsone, which has a hydroxy group at the 2 positions should be more effective than plumbagin. However, what we observed is just the opposite,—plumbagin (a 5-hydroxy-2-methyl-1, 4-naphthoquinone) effectively blocked Mtb ThyX activity, but not lawsone (a 2 hydroxy derivative). The results obtained indicate that plumbagin inhibits Mtb ThyX non-competitively with respect to dUMP. The mechanism behind this inhibition is yet to be properly understood as it would require further investigations at the structural level and the determination of the binding site.

The ability of plumbagin and lawsone to inhibit mycobacterial growth was also examined critically by evaluating their MICs for both Msm as well as Mtb. Whereas, plumbagin inhibited mycobacterial growth (MIC, 4 μg/ml, or 21.2 μM), lawsone did not. The results imply that plumbagin can function as an effective anti-mycobacterial by inhibiting ThyX. The MICs of plumbagin for Mtb and Msm were found to be the same (4 μg/ml). But in the case of rifampicin, the MICs were very different. Mtb was found to be much more sensitive (MIC <0.125), as compared to Msm (2 μg/ml). Earlier studies have also indicated that Mtb is more susceptible to rifampicin compared to Msm, possibly because it lacks an enzyme that can modify it and reduce its potency [26,27]. The MIC values obtained, in the case of Msm using the Agar diffusion assays, were about 4–5 times less as compared to those that were obtained from the broth-based experiments. The reason why the values differed is most likely because the methodologies used were different. However, we note that in both the assays, the fold difference between the MIC values of rifampicin and plumbagin was the same, the former being about half that of the latter.

We have also demonstrated that plumbagin inhibits mycobacterial growth in a dose dependent manner and that in the cells that have stopped growing, the [dTTP]/[dATP] ratio is significantly lowered. The observation is certainly a specific one, as a similar decrease in the level of dTTP relative to dATP was not observed when we used the unrelated anti-mycobacterial drug rifampicin. The conclusion that a lowering in the level of dTTP is the primary reason behind plumbagin induced cell death, is supported by the observation that the loss of viability could be reversed by overexpressing Mtb *thyX*. Interestingly, when we tried to assess the effect of *thyX* overexpression using optical density as the output, no significant difference was observed. The results indicate that plumbagin is most likely to be functioning through two mechanisms, one of which leads to growth arrest and the other which induces cell death resulting in loss of viability. The latter mode of action is most certainly caused by the inhibition of ThyX. How plumbagin produces the bacteriostatic effect is unclear, although we note that this effect is not ThyX dependent. We are, however, tempted to speculate that bacteriostasis is related to Reactive Oxygen Species (ROS) production by plumbagin. Interestingly, we found that the ROS producing activity of plumbagin is not influenced by ThyX levels (S6 Fig) and therefore ROS mediated and ThyX dependent inhibitions are possibly two different phenomena.

The present investigation is an example of how the study of mycobacteriophages can catalyze the process of identification of anti-mycobacterials. The approach that we have taken up is very similar to that adopted in an early study using staphylococcus phages which was nicknamed ‘phage inspired antibiotics’ [29,30].

## Supporting information

S1 Fig 12% SDS-PAGE analysis of purified hexa-histidine tagged Mtb ThyX protein under native condition. (PDF)

S2 Fig Box and whisker plot representation of the signal intensities obtained from mass spectroscopic analysis of dNTPs extracted from cells which were either untreated (0) or treated with 5,10,12.5 and 15 μg/ml of either plumbagin (PG, series A) or rifampicin (RIF, series B). The data used for creating these plots were derived from five independent experiments. The same data set was used to calculate [dTTP]/[dATP] ratios and their means ± SD as shown in Fig 4C and 4F of the main text.
(PDF)

S3 Fig Expression of Mtb ThyX gene in Msm using a recombinant plasmid based on the mycobacterial expression vector pLAM. We used a differential proteomics based approach to quantify the extent to which Mtb ThyX accumulates in Msm cells expressing a recombinant version of the gene. The cells were harvested by centrifugation, followed by suspension in 4 ml of Tris (50 mM) and then disrupted using a French Press. The lysate was centrifuged followed by dialysis of the supernatant against 50 mM Tris HCl. The dialysate was lyophilised and resuspended in Ammonium bicarbonate (ABC) (100 mM) solution. For protein digestion about 4 mg protein was taken in 100 μl of ABC solution and processed for trypsinization as per standard procedure [31]. The tryptic digests of the extracts were analysed by performing Liquid Chromatography Mass Spectroscopy (LCMS) using a Waters XeVo G2 XS QTof. The analysis was done using Proteomics MSEScan from 0 to 60 minutes. Peptides corresponding to Mtb ThyX were detected using the Progenesis Q1 software provided with the equipment. The results are presented for two experiments performed independent of each other on two different days.
(PDF)

S4 Fig Compilation of the relative optical densities (the optical densities of samples untreated with plumbagin were given the arbitrary value of 1) obtained 24 hrs. After the addition of increasing concentration of plumbagin, within the cells carrying either empty vector, or expressing Mtb ThyX, with (induced) or without (uninduced) acetamide induction.
(PDF)

S5 Fig Substrate saturation experiments of methylenetetrahydrofolate and NADPH, were performed using tritium release assay. Curve fitting and derivation of K_m_ and V_max_ were done using the Michelis-Menten equation with the help of GraphPad Prism software. The concentrations at which these co-substrates NADPH and methylenetetrahydrofolate support half maximal velocity (K_m_) are 35.22 and 6 μM respectively.
(PDF)

S6 Fig ROS production induced by plumbagin treatment of cells expressing the ThyX gene either at a basal (uninduced) or induced level. The population (%) of cells producing ROS was measured by performing FACS analysis after staining the cells with the fluorescent dye Dihydroethidium (DHE). Fluorescence was measured using laser settings corresponding to the Propidium iodide (PI) channel. The percentage of cells (mean +/- SD of three experiments) that are present in the DHE positive zone was plotted against the concentration of plumbagin.
(PDF)

S1 Table Viable counts following plumbagin (PG) treatment of Msm cells expressing either no gene (empty vector) or Mtb thyX (with or without induction). (PDF)
