# Supplementary material for: Mycobacterium tuberculosis thymidylate synthase (ThyX) is a target for plumbagin, a natural product with antimycobacterial activity
Source: PLoS One. 2020 Feb 4;15(2):e0228657. doi: 10.1371/journal.pone.0228657 (PMC6999906; doi:10.1371/journal.pone.0228657)
Supplement: S1 Fig — (PDF) [file pone.0228657.s001.pdf]

FIG S1

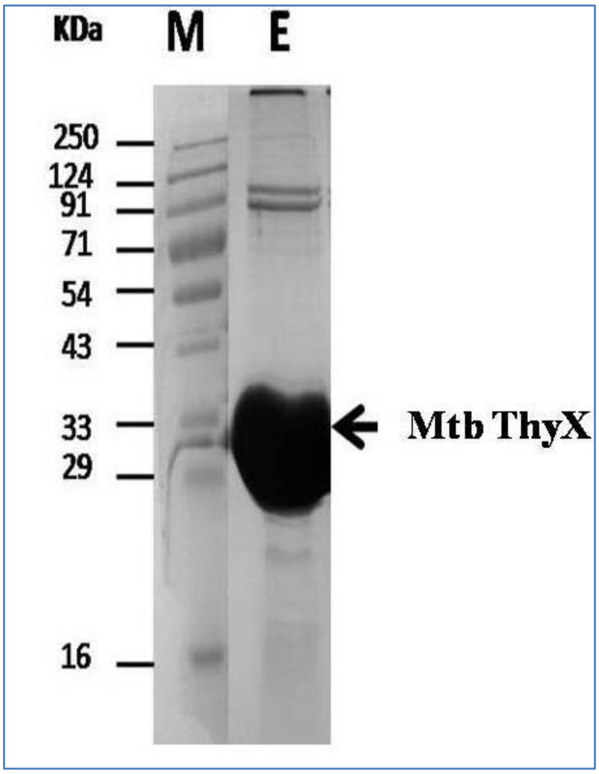

Figure S1. 12% SDS-PAGE analysis of purified hexa-histidine tagged Mtb ThyX protein under native condition.
