# Supplementary material for: Mycobacterium tuberculosis thymidylate synthase (ThyX) is a target for plumbagin, a natural product with antimycobacterial activity
Source: PLoS One. 2020 Feb 4;15(2):e0228657. doi: 10.1371/journal.pone.0228657 (PMC6999906; doi:10.1371/journal.pone.0228657)
Supplement: S2 Fig — Box and whisker plot representation of the signal intensities obtained from mass spectroscopic analysis of dNTPs extracted from cells which were either untreated (0) or treated with 5,10,12.5 and 15 μg/ml of either plumbagin (PG, series A) or rifampicin (RIF, series B). The data used for creating these plots were derived from five independent experiments. The same data set was used to calculate [dTTP]/[dATP] ratios and their means ± SD as shown in Fig 4C and 4F of the main text. (PDF) [file pone.0228657.s002.pdf]

FIG S2

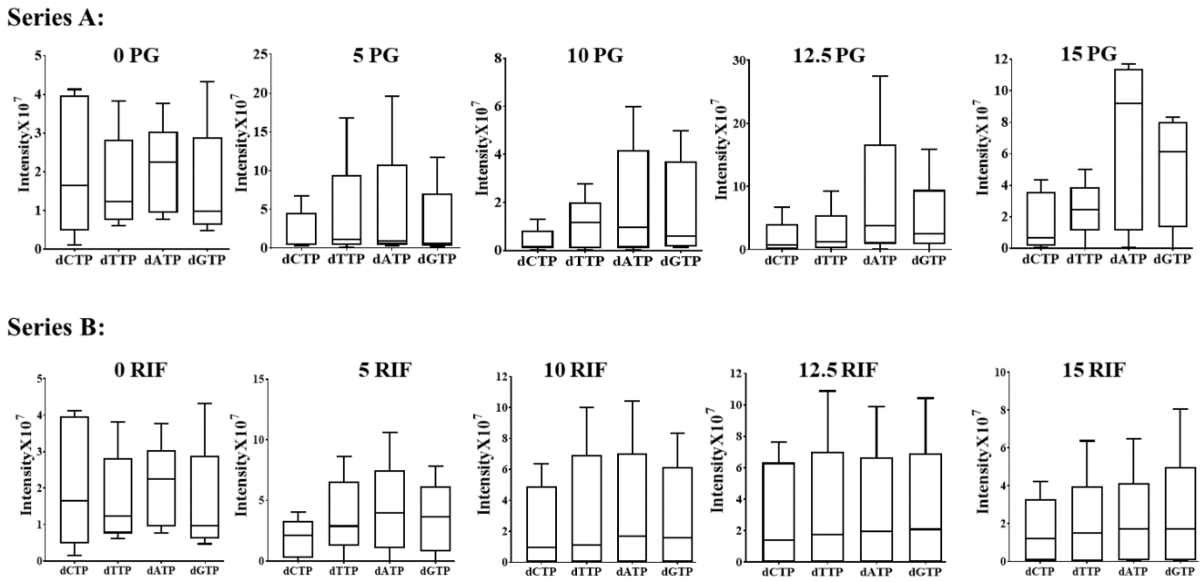

Fig. S2. Box and whisker plot representation of the signal intensities obtained from mass spectroscopic analysis of dNTPs extracted from cells which were either untreated (0) or treated with 5,10,12.5 and 15 μg/ml of either plumbagin (PG, series A) or rifampicin (RIF, series B). The data used for creating these plots were derived from five independent experiments. The same data set was used to calculate [dTTP]/[dATP] ratios and their means ± SD as shown in (Figs. 4c and f of the main text).
