# Supplementary material for: Mycobacterium tuberculosis thymidylate synthase (ThyX) is a target for plumbagin, a natural product with antimycobacterial activity
Source: PLoS One. 2020 Feb 4;15(2):e0228657. doi: 10.1371/journal.pone.0228657 (PMC6999906; doi:10.1371/journal.pone.0228657)
Supplement: S3 Fig — We used a differential proteomics based approach to quantify the extent to which Mtb ThyX accumulates in Msm cells expressing a recombinant version of the gene. The cells were harvested by centrifugation, followed by suspension in 4 ml of Tris (50 mM) and then disrupted using a French Press. The lysate was centrifuged followed by dialysis of the supernatant against 50 mM Tris HCl. The dialysate was lyophilised and resuspended in Ammonium bicarbonate (ABC) (100 mM) solution. For protein digestion about 4 mg protein was taken in 100 μl of ABC solution and processed for trypsinization as per standard procedure [31]. The tryptic digests of the extracts were analysed by performing Liquid Chromatography Mass Spectroscopy (LCMS) using a Waters XeVo G2 XS QTof. The analysis was done using Proteomics MSEScan from 0 to 60 minutes. Peptides corresponding to Mtb ThyX were detected using the Progenesis Q1 software provided with the equipment. The results are presented for two experiments performed independent of each other on two different days. (PDF) [file pone.0228657.s003.pdf]

FIG S3

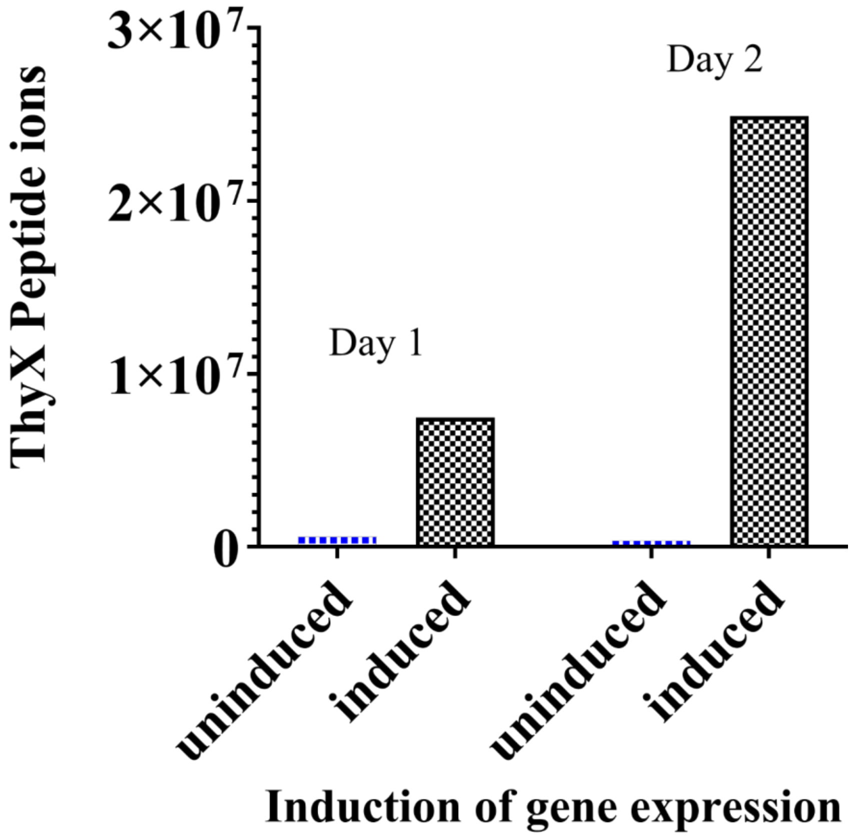

Fig. S3.

Expression of Mtb ThyX gene in Msm using a recombinant plasmid based on the mycobacterial expression vector pLAM. We used a differential proteomics based approach to quantify the extent to which Mtb ThyX accumulates in Msm cells expressing a recombinant version of the gene. The cells were harvested by centrifugation, followed by suspension in 4 ml of Tris (50 mM) and then disrupted using a French Press. The lysate was centrifuged followed by dialysis of the supernatant against 50 mM Tris HCl. The dialysate was lyophilised and resuspended in Ammonium bicarbonate (ABC) (100 mM) solution. For protein digestion about 4 mg protein was taken in 100 µl of ABC solution and processed for trypsinization as per standard procedure [23]. The tryptic digests of the extracts were analysed by performing Liquid Chromatography Mass Spectroscopy (LCMS) using a Waters XeVo G2 XS QTof. The analysis was done using Proteomics MSEScan from 0 to 60 minutes. Peptides corresponding to Mtb ThyX were detected using the Progenesis Q1 software provided with the equipment. The results are presented for two experiments performed independent of each other on two different days.
