# Supplementary material for: Mycobacterium tuberculosis thymidylate synthase (ThyX) is a target for plumbagin, a natural product with antimycobacterial activity
Source: PLoS One. 2020 Feb 4;15(2):e0228657. doi: 10.1371/journal.pone.0228657 (PMC6999906; doi:10.1371/journal.pone.0228657)
Supplement: S4 Fig — After the addition of increasing concentration of plumbagin, within the cells carrying either empty vector, or expressing Mtb ThyX, with (induced) or without (uninduced) acetamide induction. (PDF) [file pone.0228657.s004.pdf]

FIG S4

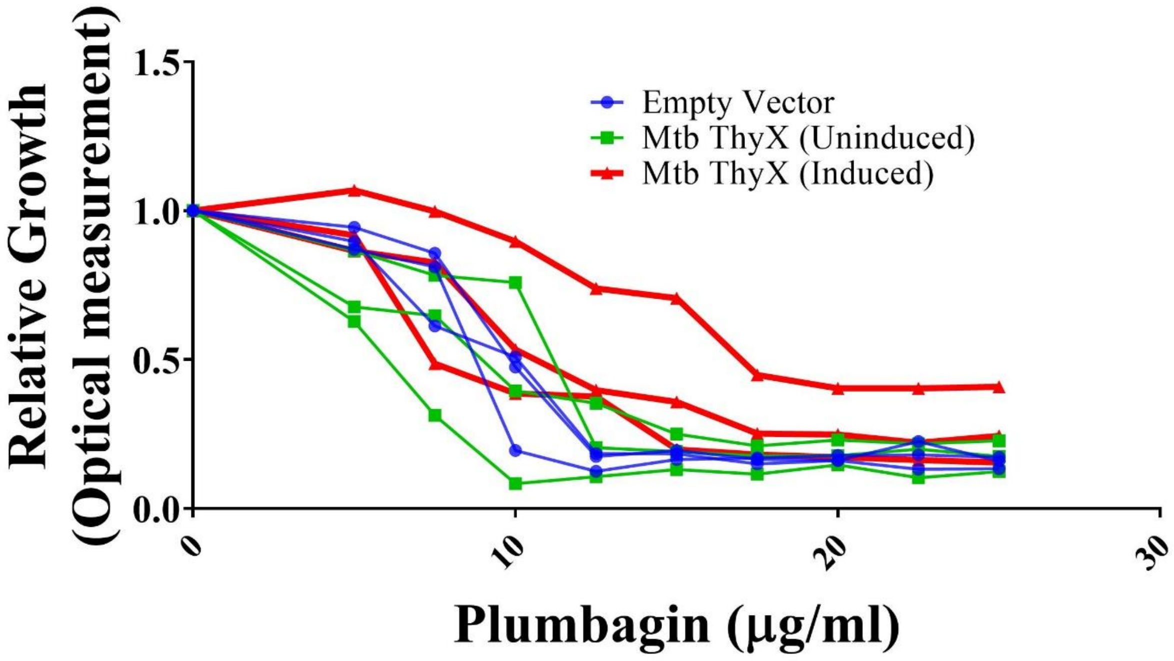

Fig. S4. Compilation of the relative optical densities (the optical densities of samples untreated with plumbagin were given the arbitrary value of 1) obtained 24 hrs. after the addition of increasing concentration of plumbagin, within the cells carrying either empty vector, or expressing Mtb ThyX, with (induced) or without (uninduced) acetamide induction.
