# Supplementary material for: Mycobacterium tuberculosis thymidylate synthase (ThyX) is a target for plumbagin, a natural product with antimycobacterial activity
Source: PLoS One. 2020 Feb 4;15(2):e0228657. doi: 10.1371/journal.pone.0228657 (PMC6999906; doi:10.1371/journal.pone.0228657)
Supplement: S5 Fig — Curve fitting and derivation of Km and Vmax were done using the Michelis-Menten equation with the help of GraphPad Prism software. The concentrations at which these co-substrates NADPH and methylenetetrahydrofolate support half maximal velocity (Km) are 35.22 and 6 μM respectively. (PDF) [file pone.0228657.s005.pdf]

FIG S5

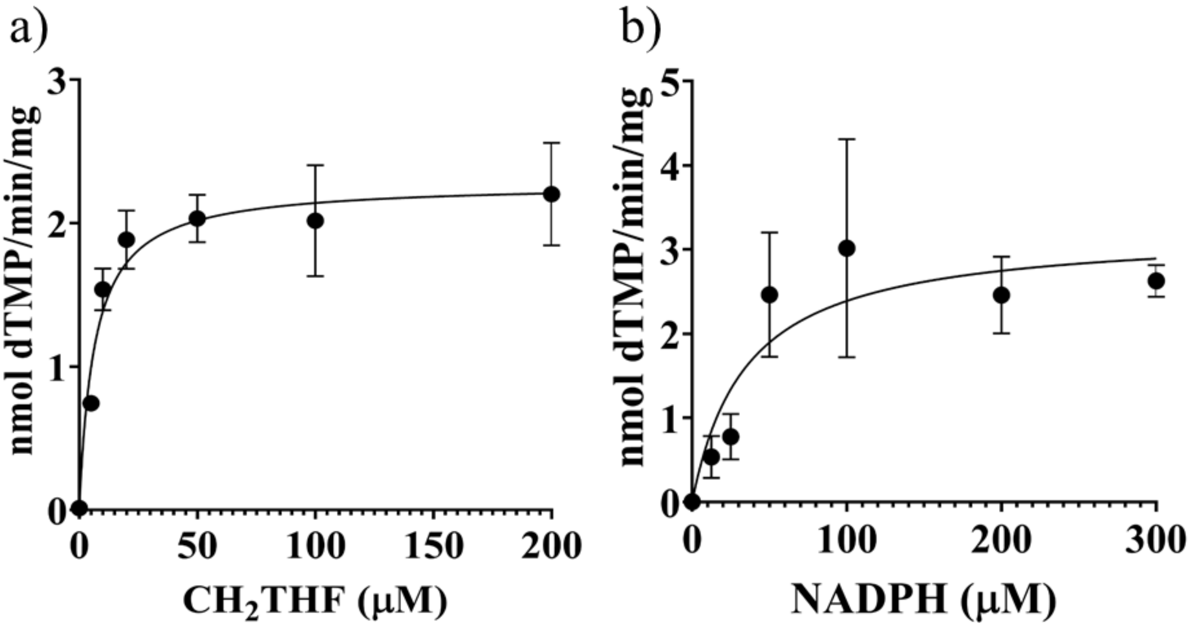

Fig. S5. Substrate saturation experiments of methylenetetrahydrofolate and NADPH, were performed using tritium release assay. Curve fitting and derivation of K_m and V_{max} were done using the Michelis-Menten equation with the help of GraphPad Prism software. The concentrations at which these co-substrates NADPH and methylenetetrahydrofolate support half maximal velocity (K_m) are 35.22 and 6 µM respectively.
