# Supplementary material for: Mycobacterium tuberculosis thymidylate synthase (ThyX) is a target for plumbagin, a natural product with antimycobacterial activity
Source: PLoS One. 2020 Feb 4;15(2):e0228657. doi: 10.1371/journal.pone.0228657 (PMC6999906; doi:10.1371/journal.pone.0228657)
Supplement: S6 Fig — The population (%) of cells producing ROS was measured by performing FACS analysis after staining the cells with the fluorescent dye Dihydroethidium (DHE). Fluorescence was measured using laser settings corresponding to the Propidium iodide (PI) channel. The percentage of cells (mean +/- SD of three experiments) that are present in the DHE positive zone was plotted against the concentration of plumbagin. (PDF) [file pone.0228657.s006.pdf]

FIG S6

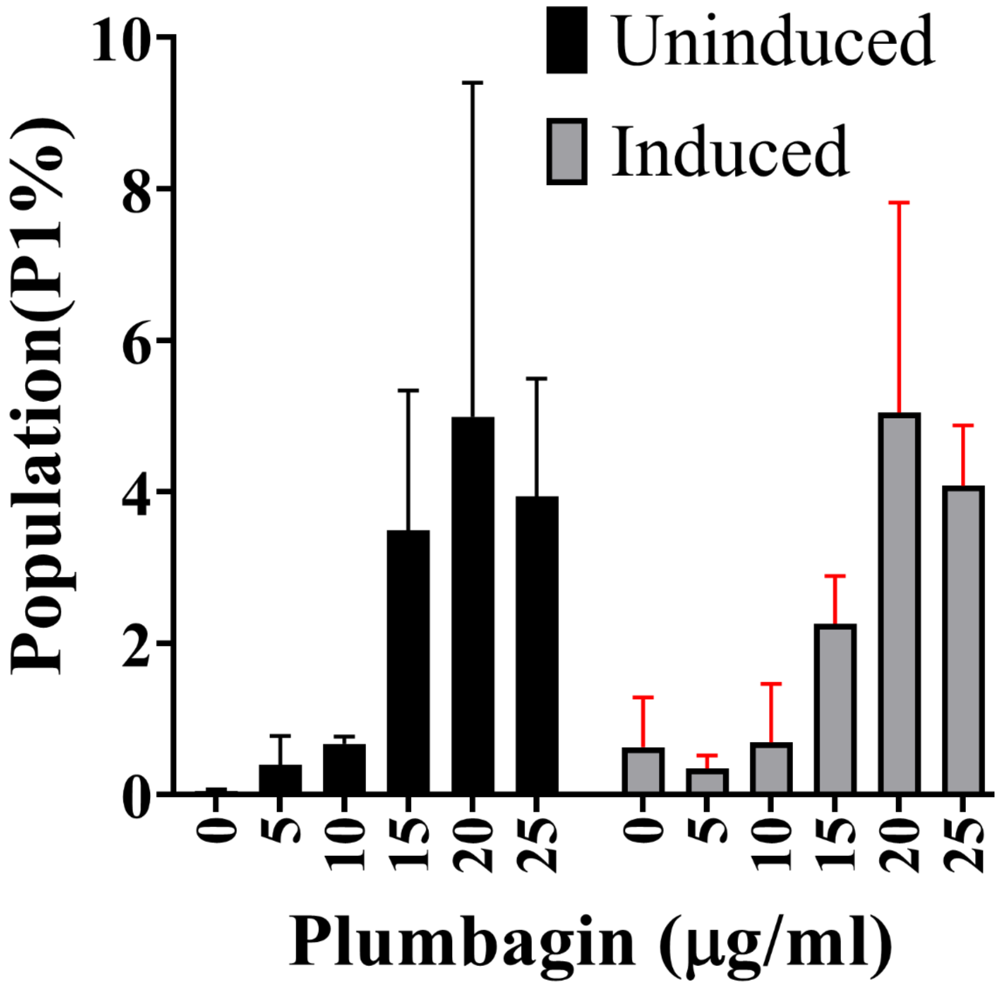

Fig. S6. ROS production induced by plumbagin treatment of cells expressing the ThyX gene either at a basal (uninduced) or induced level. The population (%) of cells producing ROS was measured by performing FACS analysis after staining the cells with the fluorescent dye Dihydroethidium (DHE). Fluorescence was measured using laser settings corresponding to the Propidium iodide (PI) channel. The percentage of cells (mean +/- SD of three experiments) that are present in the DHE positive zone was plotted against the concentration of plumbagin.
