# Supplementary material for: Mycobacterium tuberculosis thymidylate synthase (ThyX) is a target for plumbagin, a natural product with antimycobacterial activity
Source: PLoS One. 2020 Feb 4;15(2):e0228657. doi: 10.1371/journal.pone.0228657 (PMC6999906; doi:10.1371/journal.pone.0228657)
Supplement: S1 Table — (PDF) [file pone.0228657.s007.pdf]

Table S1. Viable counts following plumbagin (PG) treatment of Msm cells expressing either no gene (empty vector) or Mtb thyX (with or without induction).

PG(µg/ml)	CFUs/ml								
	Empty Vector			Uninduced			Induced		
	1	2	3	1	2	3	1	2	3
0	941 x 10^6	600 x 10^6	263 x 10^6	1021 x 10^6	650 x 10^6	363 x 10^6	532 x 10^6	200 x 10^6	186 x 10^6
5	676 x 10^6	700 x 10^6	195 x 10^6	5220 x 10^5	166 x 10^6	242 x 10^6	431 x 10^6	101 x 10^6	120 x 10^6
10	310 x 10^6	223 x 10^6	200 x 10^4	1356 x 10^5	65 x 10^6	<1 x 10^3	450 x 10^6	98 x 10^6	57 x 10^6
15	<1 x 10^3	450 x 10^2	<1 x 10^3	570 x 10^2	86 x 10^3	<1 x 10^3	94 x 10^6	56 x 10^6	43 x 10^6
20	<1 x 10^3	560 x 10^2	<1 x 10^3	196 x 10^3	18 x 10^3	<1 x 10^3	44 x 10^6	16 x 10^6	34 x 10^6
25	<1 x 10^3	103 x 10^3	<1 x 10^3	1 x 10^3	<1 x 10^3	<1 x 10^3	74 x 10^6	13 x 10^4	11 x 10^6
